# Evaluation of the academic achievements of clinician health services research scientists involved in “pre-K” career development award programs

**DOI:** 10.1017/cts.2021.780

**Published:** 2021-04-16

**Authors:** Erin F. Barreto, Rozalina G. McCoy, Joseph J. Larson, Rahma M. Warsame, Cassie C. Kennedy, Ashley E. Baker, Elizabeth S. Hart, Stephanie M. Pagel, Samantha A. Whitman, Kasey R. Boehmer, Felicity T. Enders

**Affiliations:** 1 Department of Pharmacy, Mayo Clinic, Rochester, MN, USA; 2 Robert D. and Patricia E. Kern Center for the Science of Health Care Delivery, Mayo Clinic, Rochester, MN, USA; 3 Division of Community Internal Medicine, Department of Medicine, Mayo Clinic, Rochester, MN, USA; 4 Division of Health Care Policy & Research, Department of Health Sciences Research, Mayo Clinic, Rochester, MN, USA; 5 Division of Biomedical Statistics and Informatics, Mayo Clinic, Rochester, MN, USA; 6 Division of Hematology, Department of Medicine, Mayo Clinic, Rochester, MN, USA; 7 Division of Pulmonary Medicine, Department of Medicine, Mayo Clinic, Rochester, MN, USA; 8 School for the Future of Innovation in Society, Arizona State University, Tempe, AZ, USA; 9 Knowledge and Evaluation Research (KER) Unit, Mayo Clinic, Rochester, MN, USA

**Keywords:** Translational science, education, fellowship, mentoring, publication, health services research

## Abstract

**Introduction:**

Research career development awards (CDAs) facilitate development of clinician-scientists. This study compared the academic achievements of individuals in a structured institutional “pre-K” CDA program, the Mayo Clinic Kern Scholars program, with individuals who applied for but were not admitted to the Kern program (“Kern applicants”), and awardees of other unstructured internal CDAs.

**Methods:**

This was a longitudinal cohort study of clinicians engaged in research at Mayo Clinic between 2010 and 2019. The primary outcome was time to the 15^th^ new peer-reviewed publication after the program start, adjusted for baseline number of publications. Secondarily, we described successful awarding of federal funding by the NIH or VA.

**Results:**

The median (IQR) number of baseline publications was highest among Kern Scholars compared to Kern Applicants or other CDA awardees [16 (12, 29) vs 5 (1, 11) and 8 (5, 16); P < 0.001]. After adjustment for baseline publications, the time to 15th new publication was significantly shorter for Kern Scholars than for the two comparator groups (P<0.001). Similar findings were observed with total new publications within 5 years (P < 0.001), as well as number of new first-/last-author publications within 5 years (P < 0.001). The overall frequency of K-awards, R-awards (or equivalent), or any funding were similar between groups, with the exception of R03 awards, which were significantly more common among Kern Scholars (P = 0.002).

**Conclusion:**

The Kern Scholars program is a successful training model for clinician-scientists that demonstrated comparatively greater acceleration of scholarly productivity than other internal CDA programs.

## Introduction

Rapid evolution has occurred in how healthcare is delivered, measured, and funded. There is an increased focus on translational science, quality and value, changes to payment structures, access to large datasets, and the importance of population health and preventative medicine. This has led to the development of health services research (HSR) as an essential field of scientific inquiry. HSR involves evaluation of social factors, economics, technology, organizational context, and personal behaviors that impact healthcare quality, cost, and experience. Clinician-scientists with expertise in HSR (clinician-HSR scientists) are needed to meet these demands for knowledge generation, dissemination, and evaluation [1–4].

Despite the need for clinician-HSR scientists, fewer individuals are pursuing this career path [5]. A dearth of clinician-HSR scientists is especially prominent among clinicians in surgical subspecialties [6], women [7–9], nonphysician clinician-scientists [10–12], and underrepresented minorities [9], although these gaps are not unique to HSR. To ensure a sustainable future for the advancement of medicine and its delivery, it is incumbent on the field to identify individual- and organizational-level factors that facilitate the training, persistence, and success of developing clinician-HSR scientists [3].

One successful strategy to facilitate clinician-HSR scientist development is through mentored career development awards (CDAs). Among the most sought after are the K-series awards from the National Institutes of Health (NIH), which integrate career development and applied research experience [13]. Although K-series and other comparable awards are often designed for early stage investigators, these competitive awards may still be beyond the reach of junior faculty or clinicians-engaged-in-research without an explicit academic trajectory. In these cases, alternative training pathways and funding mechanisms are necessary. Such “pre-K” CDAs may be funded by the institution, a professional society, or an NIH Clinical and Translational Science Award (CTSA) [14]. These “pre-K” programs vary in duration, breadth, and formality, but generally include some core elements of education, mentorship, and applied research experience. There is limited empirical evidence regarding programmatic features of the “pre-K” CDAs that favorably impact the success of clinician-scientists, particularly those in HSR. Previously identified factors that predict a successful transition to research independence for CDA awardees include development of robust networks for collaboration, mentorship, and institutional resources and support [15].

Mayo Clinic, an academic integrated healthcare delivery system, has several “pre-K” CDAs available for early career clinical faculty. The Mayo Clinic Kern Scholars Program is one such “pre-K” CDA focused on the development of the clinician-HSR scientist. The Kern Scholars program seeks to develop individuals who could transform health care delivery through rigorous, collaborative, practice-oriented research. The program includes two tracks. The faculty track lasts 2–3 years, provides for up to 40% protected research time, and offers modest research funding (Table S1). The fellow track provides for one year of 90% protected research time and comparable yearly research funding.

**Fig. 1.**
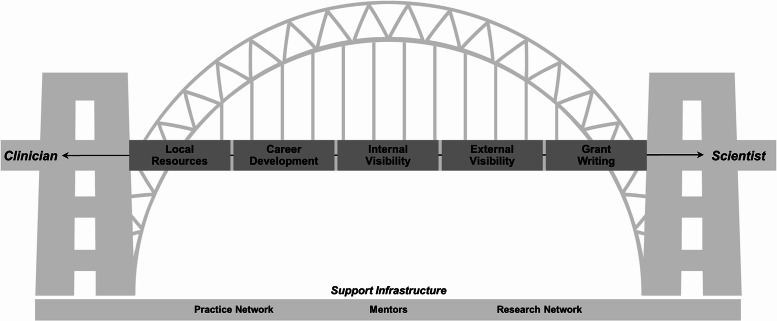
Conceptual framework for developing the bridge between clinician and scientist. Key domains include career development, internal networking and visibility, external networking and visibility, a familiarity with local resources, and expertise in grant writing. The practice and research networks as well as mentors provide necessary infrastructure to support the development of clinician-health services research scientists.

The Kern Scholars program includes several key elements to facilitate a candidate’s successful development as a clinician-HSR scientist (Table S2). Peer coaching is based on a cascading mentorship model, where senior scholars provide mentorship to junior scholars [16–18]. A physical space within the Kern Center exists to directly facilitate collegiality, engagement with core faculty and resources, and peer coaching. In the 1–3-year-award period, scholars meet weekly for structured “works-in progress” sessions, which include scholar presentations, invited faculty lectures, and career development topics. Each scholar presents their research during this interactive session approximately every six months. As with many elements of the program, works-in-progress sessions are tailored to the needs of the scholars as appropriate for their career stage and ongoing research activities. Examples of topics presented include a research project that encountered obstacles (e.g., slow enrollment, study personnel challenges), a specific aims page or draft of a grant in need of revision, or preliminary data for summary and interpretation. In addition to scholar-led works-in-progress, focused career development training occurs at monthly intervals and during biannual one day retreats. Selected topics transcend the traditional scientific areas in a deliberate attempt to provide a holistic approach to career development. Topics were also chosen to develop clinician-HSR scientists able to build bridges [3,4] across research’s “Valley of Death [19],” i.e., the gap between promising discoveries and bedside treatment (Fig. 1).

Other internal “pre-K” CDA programs (i.e., benefactor-funded CDAs) exist at Mayo Clinic, which provide up to $50,000 to $100,000 per year over one to three years that may be used to support protected time or other research support (Table S1). Mayo Clinic offers approximately 20 benefactor funded “pre-K” CDAs per year across various disease-state and topical subspecialties. These other internal “pre-K” CDA programs use a traditional cognitive apprentice mentoring model, which is predicated on one individual (generally the principal investigator) mentoring one or more learners [16–18]. Each recipient is also separate from other awardees without group activities, so there is less of an opportunity for a cohort effect or peer-to-peer mentorship. Informal interactions with colleagues and mentors not dictated by the award mechanism are expected consistent with usual practice. In addition, these other “pre-K” CDA awardees do not participate in a structured curriculum, though some may choose to utilize a portion of their funding for individualized scientific education (i.e., coursework, conferences, workshops). These distinct “pre-K” training models offer an opportunity for comparison.

In this report, we compare the academic output of Mayo Clinic Kern Scholars to other internal benefactor-funded “pre-K” CDAs. We hypothesized that Kern Scholars would experience greater academic success (measured with peer-reviewed publications and grant funding) than other career development awardees over the same time due to the program’s structured curriculum.

## Methods

### Setting and Participants

We conducted a longitudinal cohort study that compared scholarly productivity and grant funding among clinicians at three Mayo Clinic campuses (Minnesota, Florida, Arizona) who were classified into three groups between 2010 and 2019: (1) Kern Scholar awardees in the faculty track, (2) Kern Scholar applicants who applied but were not accepted, and (3) recipients of other Mayo Clinic benefactor-funded CDAs. The Mayo Clinic Institutional Review Board approved the protocol as exempt (IRB 19-006187).

Fellows selected for the Kern Scholars program were excluded due to lack of appropriate comparators (N=7). No fellows were included in the other study arms. Individuals selected for both the Kern Scholars program and an internal “pre-K” CDA were handled in two ways. When these two “pre-K” CDAs were granted in the same year, individuals were excluded (N=4) as we were unable to discern the timeline of productivity relative to the award. If the awards were granted in different years (e.g., Kern Scholars acceptance in year 1, benefactor-funded CDA in year 3), individuals were grouped according to the first award received and censored at the start of the second program. The index date was the year of application or entry into the program. Given the variable duration of each program, all individuals were followed until June 2019 (end of the study period). Individuals whose publications and grants could not be characterized through publicly available medical literature databases (i.e., PubMed) or funding agency portals were also excluded (N=6). The Mayo Clinic Institutional Review Board approved the protocol as exempt (IRB 19-006187).

### Outcomes

The primary outcome of interest was academic achievement as measured by time to the 15^th^ new peer-reviewed publication (i.e., manuscript published after the index date) [20] and the number of first author publications, last author publications, or first-/last-author publications between index date and five years of follow-up or the end of study period. Peer-reviewed publications were identified through an institutionally managed database (RE-AIMS) for individuals currently at Mayo Clinic and reconciled with publicly available medical literature databases (i.e., PubMed). Secondarily, we evaluated achievement of federal funding by the CDA awardee as a primary investigator. Federal funding was identified using two publically available databases from the NIH https://projectreporter.nih.gov/reporter.cfm) or the Veterans Administration (https://www.hsrd.research.va.gov/research/cda.cfm). K-, R-, U- and P-awards were counted toward this outcome whereas F32 and F30 awards were not. For the Kern Scholars, we also described all non-institutional (i.e., federal, foundation, or industry) research funding during their time at Mayo Clinic sourced from an internal data repository. This information (foundation and industry awards) was not available for the other two groups. Exploratory analyses in women, underrepresented minorities, and those affiliated with surgical subspecialties were performed to determine differential success within these subgroups. The H-index [21] was also retrospectively calculated for all individuals at the index date and at the date of last follow-up (June 2019). The H-index integrates publication and citation counts to generate a summary measure of scientific achievement for an individual researcher [21]. The intra-individual change in H-index from index date to the end of the study period was described.

### Analyses

Descriptive comparisons between Kern Scholars, Kern applicants (not selected), and other CDA awardees used means with standard deviations (SD) or medians with interquartile ranges (IQR), and frequencies with percentages, as appropriate. Time to 15^th^ new publication after the index date was examined using Kaplan Meier survival analysis. Comparisons across the three groups were made with the log-rank test. Univariate Cox-models were fit for time to 15^th^ new publication, first author publication, last author publication, or first-/last-author publication. Models were then adjusted for the number of publications prior to the index date. To assess pairwise differences between the Kern Scholars and other comparator groups in the number of new publications within five years of the index date, quasi-Poisson regressions models were fit. The quasi-Poisson model was chosen to address overdispersion due to the proportion of individuals with zero publications within the five years of index date. Univariate analyses were performed as well as analyses adjusted for number of publications prior to the index date. The presence or absence of funding at the conclusion of the study timeframe was treated as a binary variable and analyzed with the Chi-square or Fisher’s Exact test. A P-value <0.05 was considered statistically significant. All analyses were conducted using the R (v3.6.2, Vienna, Austria) statistical package.

## Results

**Fig. 2.**
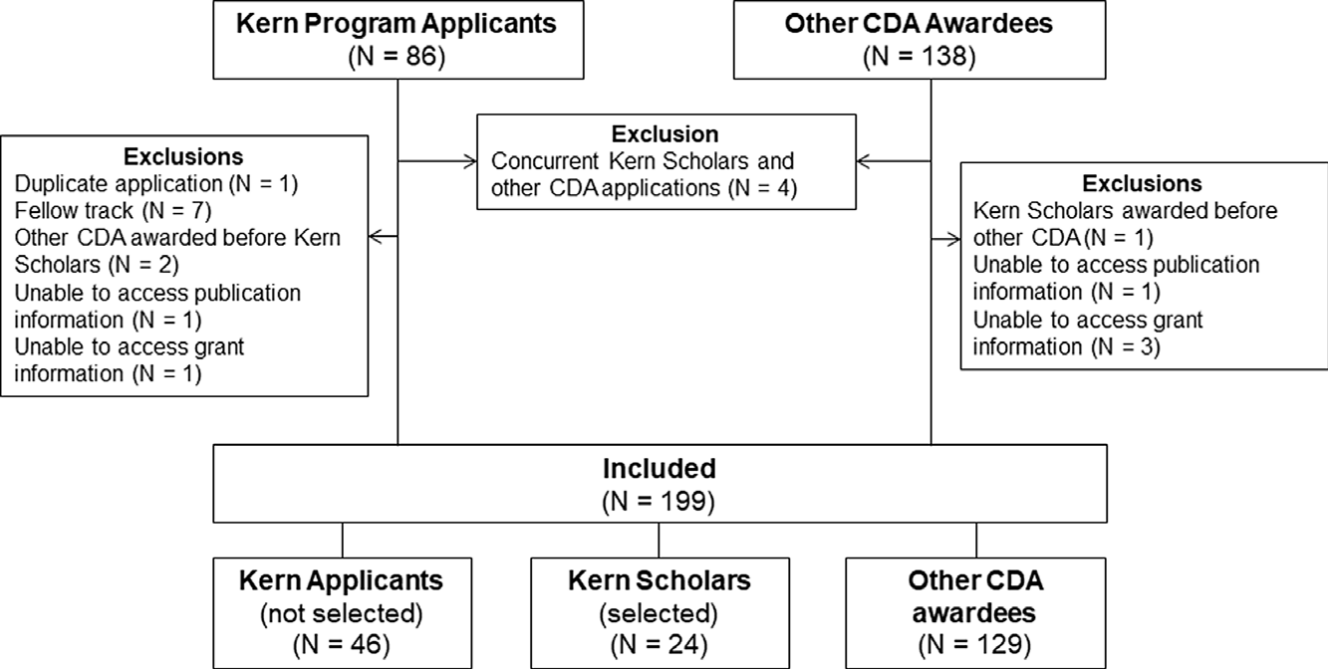
Study flow diagram CDA, Career development award.

During the study period, there were 46 Kern applicants (not selected), 24 Kern Scholars, and 129 other CDA awardees included in the analytic cohort (Fig. 2). Demographic characteristics of individuals in each group are summarized in Table 1. At baseline, the median (IQR) total number of peer-reviewed publications at the time of program enrollment/application was higher for Kern Scholars than Kern applicants [16 (12, 29) vs 5 (1, 11); P < 0.001] and for Kern Scholars than other CDA awardees [16 (12, 29) vs 8 (5, 16); P < 0.001]. Numbers of first author publications and first-/last-author publications were different across groups (Table 1). The H-index was able to be calculated for 191 (96%) individuals. Kern Applicants had the lowest H-index at baseline at 6 (3.5, 14.0), compared to 10 (7.4, 13.8) in Kern Scholars, and 13 (8.3, 20.0) in other CDA awardees (P < 0.001).

**Table 1.** Characteristics of the study cohort

Characteristic	Overall (N = 199)	Kern Applicants^a^ (N = 46)	Kern Scholars (N = 24)	Other CDA awardees (N = 129)	P-Value
Demographics (N; %)					
Female sex^b^	74 (39)	16 (36)	12 (50)	46 (37)	0.47
Department^c^					<0.001
Nonsurgical	153 (78)	35 (76)	21 (88)	97 (77)	
Surgical	22 (11)	3 (7)	2 (8)	17 (14)	
Allied health	3 (2)	1 (2)	1 (4)	1 (1)	
Other	10 (5)	0 (0)	0 (0)	10 (8)	
Unknown	8 (4)	7 (15)	0 (0)	1 (1)	
Race/ethnicity^d^					0.38
White	115 (59)	24 (52)	16 (67)	75 (60)	
Asian	53 (27)	14 (30)	3 (13)	36 (29)	
Black/African American	10 (5)	4 (9)	2 (8)	4 (3)	
Hispanic or Latino	11 (6)	2 (4)	2 (8)	7 (6)	
Other/Unknown	6 (3)	2 (4)	1 (4)	3 (2)	
Program year (N; %)					0.004
2010	5 (3)	0 (0)	0 (0)	5 (4)	
2011	11 (6)	0 (0)	4 (17)	7 (5)	
2012	21 (11)	3 (7)	2 (8)	16 (12)	
2013	15 (8)	4 (9)	4 (17)	7 (5)	
2014	28 (14)	12 (26)	4 (17)	12 (9)	
2015	24 (12)	5 (11)	2 (8)	17 (13)	
2016	28 (14)	9 (20)	1 (4)	18 (14)	
2017	25 (13)	7 (15)	5 (21)	13 (10)	
2018	23 (11)	6 (13)	3 (8)	15 (12)	
2019	19 (10)	0 (0)^e^	0 (0)^e^	19 (15)	
Prior publications^f^					
Total	9 (4, 15)	5 (1, 11)	16 (12, 29)	8 (5, 16)	<0.001
First author	6 (3, 11)	4 (1, 8)	7 (4, 13)	7 (3, 12)	0.003
Last author	1 (0, 3)	0 (0, 2)	1 (0, 5)	1 (0, 4)	0.16
First or last author	8 (4, 14)	5 (1, 11)	9 (6, 16)	8 (4, 16)	0.003

CDA, Career development award.

a Refers to individuals who applied to the Kern Scholars program and were not selected.

b Data were available for N = 44 Kern Scholars, N = 24 Kern Applicants, and N = 124 other CDA awardees.

c Data were available for N = 46 Kern Scholars, N = 24 Kern Applicants, and N = 126 other CDA awardees.

d Data were available for N = 46 Kern Scholars, N = 24 Kern Applicants, and N = 125 other CDA awardees.

e No Kern Scholars were included from 2019 as their program would have begun in July which post-dates the study follow-up period.

f Data represented medians with interquartile ranges prior to the index date. Two-way comparisons indicated statistically more total publications prior to the index date among Kern Scholars compared to other CDA awardees (P < 0.001). No statistical difference existed between Kern Scholars and other CDA awardees in number of first author (P = 0.30), last-author (P = 0.69), or first-/last-author publications (P = 0.56) at baseline. Kern Scholars had more baseline publications than Kern applicants in each of the prior publication categories with the exception of last author publications (P = 0.10).

### Endpoints

**Fig. 3.**
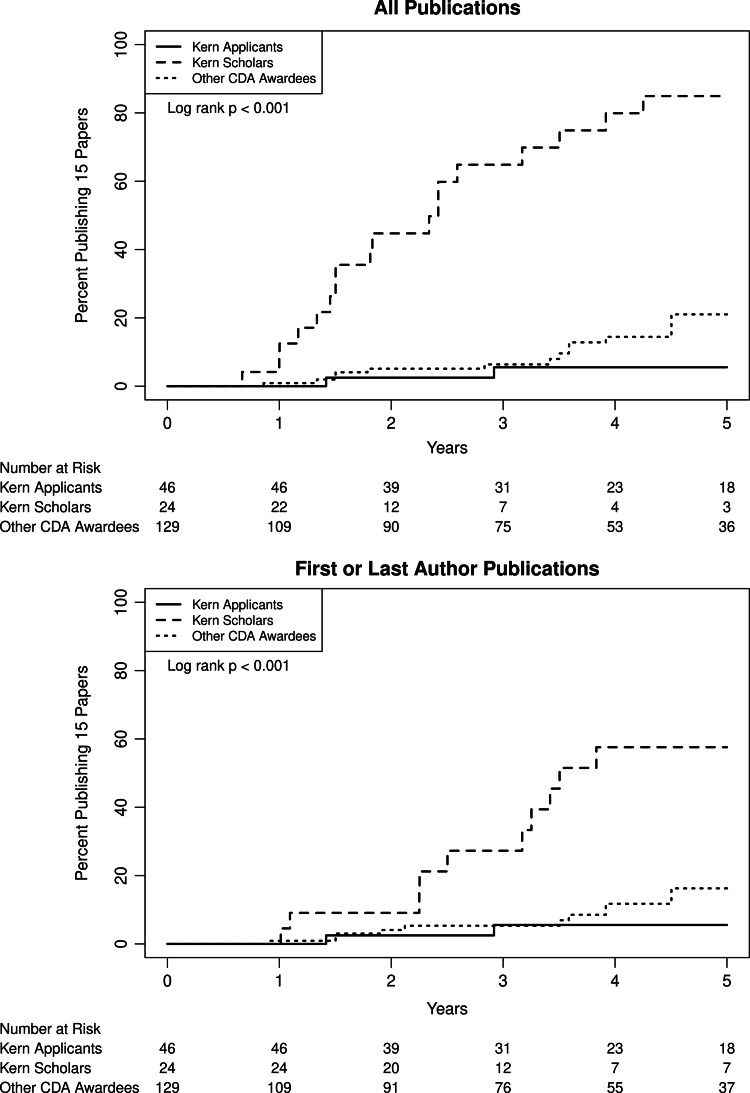
Time to 15^th^ new publication across groups. Upper panel: Time to 15^th^ new publication between Kern Scholars and Kern applicants or other CDA awardees. Lower panel: Time to 15^th^ new first or last author publication between Kern Scholars and Kern applicants or other CDA awardees. Kern Scholars achieved a more rapid time to 15^th^ new publication and 15^th^ new first-/last-author publication than comparator groups (P < 0.001 in both cases). CDA: Career development award.

Time to 15th new publication was significantly shorter for Kern Scholars than the two comparator groups (P < 0.001) (Fig. 3, upper panel). The 15th new publication occurred at a median of 2.4 (95% CI: 1.5, 3.9) years from the index date for Kern Scholars, compared to more than 8.5 years for the other two groups. By eight years, 90% of Kern Scholars had achieved 15 new publications compared to 6% and 35% in Kern Applicants and other CDA awardees, respectively. Similar findings were observed for time to 15^th^ new first-/last-author publication (P < 0.001) (Fig. 3, lower panel). After adjustment for prior publications at the index date, Kern Scholars had a significantly faster time to 15^th^ new publication and 15^th^ new first-/last-author publication compared to Kern applicants and other CDA awardees, though no difference was noted when first authorship and last authorship were evaluated separately (Table 2). The median (IQR) number of total new publications within five years was significantly different across groups [Kern Scholars 27 (17, 42) vs Kern applicants 4 (1, 7) vs other CDA awardees 4 (1, 8); P <0.001]. Similarly, the median number of first author publications was significantly different [Kern Scholars 7 (3, 10) vs Kern applicants 1 (0, 3) vs other CDA awardees 1 (0, 2); P <0.001]. After adjustment for publications prior to the index date, the total number of publications within five years was higher among Kern Scholars than the other two groups (Table S3).

**Table 2.** Cox proportional hazards models for predicting time to 15^th^ new publication based on various definitions, adjusted for publications prior to the index date

Publication category	Adjusted HR (95% CI)	P-value
Kern Scholars compared to Kern applicants		
Any publication	10.8 (2.3, 51.8)	0.003
First author publication	–^a^	–^a^
Last author publication	0.08 (0.004, 1.5)	0.089
First or last author publication	2.9 (0.5, 17.0)	0.25
Kern Scholars compared to other CDA awardees		
Any publication	9.9 (4.5, 18.9)	<0.001
First author publication	4.8 (0.4, 57.0)	0.21
Last author publication	1.7 (0.6, 4.7)	0.34
First or last author publication	3.8 (1.6, 9.0)	0.002

CDA, Career development award; HR, Hazard ratio.

a Unable to be calculated as no individuals in the Kern applicants group reached 15 new first author publications.

Overall, 48 (31%) individuals successfully secured extramural funding as a principal investigator from the NIH during the follow-up period (Kern Scholars 33% vs other CDA awardees 31%; P = 0.82). No individuals were identified with funding through the VA. No Kern applicants received this type of funding during the study timeframe. Twenty six (17%) individuals were awarded K-type grants and 35 (23%) received R-, P- or U-awards. There was no significant difference between Kern Scholars and other CDA awardees in the frequency of any funding, receipt of a K-award, or receipt of an R-, U-, or P-award as PI (Table 3). Kern Scholars were significantly more likely to receive an R03 than other CDA awardees (21% vs 4%, respectively; P = 0.002). For the 24 Kern Scholars, data on all types of noninstitutional funding were available for 18 (75%) individuals. During the study time frame, the median total funding from external sources per person was $563,874 ($63,114, $2,626,112). Median funding secured by Kern Scholars as principal investigator was $298,386 ($19,459, $1,050,655) and as coinvestigator was $56,250 ($625, $864,110).

**Table 3.** National Institutes of Health Funding as Principal Investigator

Characteristic	Overall (N = 153)^a^,^b^	Kern Scholars (N = 24)^a^	Other CDA Awardees (N = 129)^a^	P-Value
K award	26 (17)	5 (21)	21 (16)	0.59
R-, P- or U-awards	35 (23)	6 (25)	29 (23)	0.79
R01, P- or U-awards	25 (16)	3 (13)	22 (17)	0.58
R03 awards	10 (7)	5 (21)	5 (4)	0.002
R21 award	7 (5)	1 (4)	6 (5)	0.92
Other R award	4 (3)	0 (0)	4 (3)	1.00
Any funding^c^	48 (31)	8 (33)	40 (31)	0.82

CDA, Career development award.

a Values expressed as frequencies with percentages.

b No Kern applicants were awarded NIH funding during the study timeframe; thus, they are omitted from the denominator.

c Considers K-, R-, P-, and U-series awards; F awards were not included in the analysis.

### Exploratory Analyses

In stratified analyses according to sex (male vs female), race (white vs nonwhite), and specialty (surgical vs nonsurgical), the primary outcome of time to 15^th^ new publication appeared generally similar to the overall group, but statistical inferences were limited due to small sample sizes in some subgroups (Figure S1–S3). The change in H-index per year from baseline over the follow-up period was similar across groups (P = 0.21).

## Discussion

Training the next generation of clinician-HSR scientists, particularly those directly embedded in the practice, is a central priority for the medical community, health systems, and society. We describe the programmatic elements and outcomes of the Mayo Clinic Kern Scholars program, a mentored institutional “pre-K” CDA, which effectively trained 24 clinician-HSR scientists over the past ten years. We demonstrated considerable success of this program in terms of academic productivity and successful transition to extramural funding. Compared to other “pre-K” intramural CDA awardees, who received a similar amount of protected time and financial resources, Kern Scholars demonstrated a 10-fold faster time to 15^th^ new publication and a 4-fold faster time to 15^th^ new first-/last-author publication. Similar findings were observed among women, underrepresented minorities, and individuals in the surgical subspecialties. One-third of Kern Scholars and other CDA awardees secured extramural funding as a principal investigator from the NIH during the follow-up period. The comparative frequency of successful extramural funding was similar across award subtypes, with the exception of R03s, which were more common among Kern Scholars. When considering all extramural funding sources (federal, foundation, and industry), Kern Scholars received a median of $563,874 in grant funding per person.

Evidence suggests that formal training in clinical translational research promotes scholarly output. In a study of 70 clinical fellows who completed formal postdoctoral clinical translational research training, matched to fellows who did not, the number of peer-reviewed manuscripts, including first-/last-author manuscripts, and the H-index were higher among those who completed formal training [20]. While these data suggest a benefit of enhanced research training, this study did not assess whether specific elements of a training program enhance the probability of long-term success. It is expected that all individuals in the present study were clinicians interested in an academic path, given their application to these programs. The comparison between Kern Scholars and other CDA awardees allowed us to probe the influence of structured programmatic elements on candidate success. Research time and funding amounts were likely less influential on the observed outcomes as these factors were similar between groups. Using an estimated salary based on the NIH cap of approximately $200,000, Kern Scholars received $61,000 per year for three years (salary, fringe, and prorated research funds over the life of the award) compared to $50–$100,000 per year for other CDA awardees (Table S1). Detailed individual award amounts were not available. We hypothesize that one reason for greater probability of academic success among the Kern Scholars was the frequent and facilitated interactions among and between the Scholars and the program leadership, which complemented the training and mentoring from mentors. While not directly measured in this study, we surmise that weekly interactions may enhance skill building, feedback, modeling, confidence, and emotional support.

The Kern Scholars Program approach differs from the traditional dyadic model of one protégé/mentee and one senior faculty mentor, common in PhD and other research training programs [16–18]. In an integrated model such as the Kern Scholars program, emphasis is instead placed on cascading mentorship wherein junior scholars are trained by senior scholars. This horizontal mentorship or mentor network model [18,22] may be more effective in cultivating clinician-HSR scientists, as it mimics the familiar layered learning model used in medicine (i.e., hierarchical team model of medical student, resident, fellow, staff) [23] and the composition of multiprofessional collaborative clinical teams. This is aligned with data from a qualitative study of 40 former KL2 or K12 Scholars, which suggested that research networks and mentorship are key factors associated with successfully securing independent funding at the conclusion of the CDA. One respondent in this evaluation described several types of mentors necessary for academic success one of which was a peer mentor to serve as a role model and guide [15].

Internal “pre-K” CDAs are one mechanism to foster the development of clinician-HSR scientists. These awards are often designed as a bridge toward external funding via a K-type award (i.e., K08 Mentored Clinical Scientist Research CDA or K23 Mentored Patient-Oriented Research CDA) or an R-series award. In this study, 31% of individuals were awarded federal funding as PI during the follow-up interval. Seventeen percent of individuals were awarded a K-series grant. It is unknown what percentage of people in these programs submitted NIH grant applications during the follow-up period and were not funded. Estimates indicate that success rates for K08 or K23 applications for the general applicant pool range from 35 to 45% [17]. Other than federal support, possible sources of funding include from industry and foundation grants. Our data demonstrates that 71% of Kern Scholars were awarded noninstitutional funding (federal, foundation, or industry) during or after program completion. This suggests that these other sources of funding play a considerable role in the academic careers of clinician-scientists. Future studies and training programs should include industry and foundation funding as metrics for success alongside federal awards.

While these data are promising, the study has several limitations. Internal awards require substantial resources from the institution, divisions/departments, and/or benefactors and as such are heavily influenced by the environment. Evidence from one survey of academic emergency departments indicated that 27% of centers provided institutional research funds to support extramural grant applications for junior researchers [24]. Mayo Clinic is a large academic integrated health care delivery system with an extensive research program; thus, these results may not be generalizable. However, Mayo Clinic is also not a part of a larger university and as such its research training programs are directly embedded in the practice, demonstrating the possibility of successful early career research training outside of a strictly academic setting. Another consideration is that Kern Scholars may have been more academically accomplished at baseline than the other groups rendering these findings self-fulfilling. Indeed at baseline, Kern Scholars had more peer-reviewed publications than the other two groups, but the H-indices between Kern Scholars and other CDA awardees were similar at baseline. Still differences between groups in the academic trajectory were realized even after adjustment for baseline productivity. It is also possible that there is bias associated with allocation to the various groups under study. The justification for selection of a given individual for participation in any one of these programs was unobserved. Another limitation is that the time horizon during which one should measure academic success and the metrics to be used remain a subject of debate. Among other CDA awardees (not exclusively HSR), data collection for biologic/mechanistic work may require more time to complete thereby delaying publications relative to HSR scholars. In this study, we attempted to minimize this effect by extending follow-up out to at least five years. We acknowledge that not all peer-reviewed publications carry the same clinical impact, methodological rigor, or potential to elevate an early investigator’s career. To address this, we also examined first and last author publications, which reflect more active involvement from the investigator. Finally, while we performed descriptive analyses that suggest the robustness of our findings among women, underrepresented minorities, and individuals from surgical subspecialties, the small sample size in these groups limited inferences that could be made.

While our work has identified marked success for the Kern Scholars program when compared to another “pre-K” CDA training pathway, further research will be needed to determine the programmatic elements to which the success can be attributed. It is not clear whether the observed success is a function of the structured academic environment, candidate selection, added mentorship by program leaders experienced in HSR, and/or the cascading peer mentorship philosophy. We do not capture the frequency of papers coauthored by multiple Scholars/awardees, but this could signal enhanced collaboration and be a target for future investigation. It is also not clear whether some trainees may need assistance in different arenas, which could further enrich their careers. For instance, scholars are located at three sites (Minnesota, Florida, Arizona). It is likely that cascading peer mentorship requires a critical mass of individuals, but it is not clear whether it is more important for those individuals to have a common geographic location, similar research, or personal features. Ongoing qualitative assessments will address these questions. We would also like to see future research assess which productivity components are most predictive of future leadership roles. While the traditional research outcomes we assessed are critical for developing a research program, ongoing sustainability and practice integration of HSR research depend upon elevation of clinician-HSR scientists as practice leaders.

The job of the clinician-HSR scientist has simultaneously become more vital and more challenging in recent years. The pace of academic medicine is accelerating, and the breadth and scope of clinical data is increasing exponentially. The primary focus of published literature has shifted from preclinical and mechanistic studies toward public health, quality, epidemiological research [25]. Increasing clinical responsibilities and patient complexity tax the demands of already busy clinicians, diminishing motivation for a career in research [24]. With more knowledge to review, appraise, synthesize, and apply, the gap between academic research and meaningful changes to clinical practice remains broad. Development of clinician-HSR scientists is essential to address these challenges and improve patient care quality and efficiency.

## Conclusions

The approach to and impact of “pre-K’ training pathways for clinician-HSR scientists have not been extensively studied, but are critical to the development and preservation of a robust clinician-HSR scientist workforce. We found the Kern Scholars program was a successful training model for clinician-HSR scientists that demonstrated comparatively greater scholarly productivity than other internal CDA programs. Future studies will explore the first-person experience of Kern Scholars through in-depth qualitative interviews. These data will elucidate perceived strengths and limitations of the current model and opportunities for future iteration. Additional study is needed to further characterize the impact of CDA programs on other metrics of academic success including promotion and leadership roles.

## Supplementary material

For supplementary material accompanying this paper visit http://dx.doi.org/10.1017/cts.2021.780. click here to view supplementary material
