# Supplementary material for: Evaluation of the academic achievements of clinician health services research scientists involved in “pre-K” career development award programs
Source: J Clin Transl Sci. 2021 Apr 16;5(1):e122. doi: 10.1017/cts.2021.780 (PMC8278162; doi:10.1017/cts.2021.780)

**Supplementary Appendix**

**Table S1.** Features of Kern Scholars program compared to other local CDAs

| Features | Kern Scholars | Other local CDAs |
| --- | --- | --- |
| Size of cohort | 2-5 per cohort each year | 1 per award, 20+ awards per year^b^ |
| Protected time | 20-40%^a^ | Flexible, covered by total funding support |
| Length | 2-3 years^a^ | 1-3 years |
| Research funding | $20,000 total | $50,000-100,000 per year |
| CDA core elements  Didactic coursework  Peer coaching  Career development  Mentoring | Per preference  Weekly sessions  Monthly sessions  Biannual retreats  Continuous, structured | Per preference  N/A  N/A  Continuous, semi-structured |
| a: Individuals in the Fellow track of the Kern Scholars program receive 90% protected time for 1 year  b: Number of available awards varies each year, but in 2020, 23 separate awards were funded | | |

**Table S2.** Kern Scholars program details

| Program eligibility |
| --- |
| - Career clinical faculty (i.e. doctoral-trained clinicians actively engaged in clinical care) or clinical trainees (i.e. residents or fellows) - Demonstrated interest and departmental support for pursuit of a career in health services research - Not funded at the federal level as a principal investigator - Typically Assistant Professor level with 10-30 publications; if more senior or more prolific, intent to change research directions toward health services research |
| Focus areas |
| Patient-reported outcomes  Secondary data analysis  Systematic reviews and meta-analyses  Shared decision-making  Health disparities  Quality improvement  Behavioral interventions  Comparative effectiveness research  Implementation science  Informatics and knowledge management  Data science  Economic evaluation  Health systems engineering  Innovation and design  Qualitative research |
| Structured program elements |
| - Peer coaching - Weekly 1-hour career development and works-in-progress sessions - Monthly 3-hour intensive career development sessions   - Topics include: Personal and professional career development, internal networking and visibility, external networking and visibility, local resources, health services research methods, and grant writing - Mentoring by clinical and methodologic experts - Biannual 1-day retreats - Colocation in a dedicated space in the Mayo Clinic Center for the Science of Health Care Delivery |

**Table S3**. Number of publications within five years for Kern scholars compared to Kern applicants and other CDA awardees adjusted for publications prior to the index date

| Publication category | Adjusted RR (95% CI) | P-value |
| --- | --- | --- |
| *Kern Scholars compared to Kern applicants* | | |
| Any publication | 3.1 (2.0, 5.0) | <0.001 |
| First author publication | 2.5 (1.4, 4.4) | 0.002 |
| Last author publication | 1.0 (0.6, 1.9) | 0.92 |
| First or last author publication | 1.7 (1.0, 2.7) | 0.044 |
| *Kern Scholars compared to other CDA awardees* | | |
| Any publication | 4.6 (3.5, 6.0) | <0.001 |
| First author publication | 3.2 (2.2, 4.7) | <0.001 |
| Last author publication | 1.7 (1.1, 2.6) | 0.011 |
| First or last author publication | 2.2 (1.6, 3.1) | <0.001 |

**Figure S1. Time to 15^th^ new publication stratified by sex.**

**Upper panel:** Time to 15^th^ new publication between Kern Scholars and Kern applicants or other CDA awardees.

**Lower panel:** Time to 15^th^ new first-/last-author publication between Kern Scholars and Kern applicants or other CDA awardees.


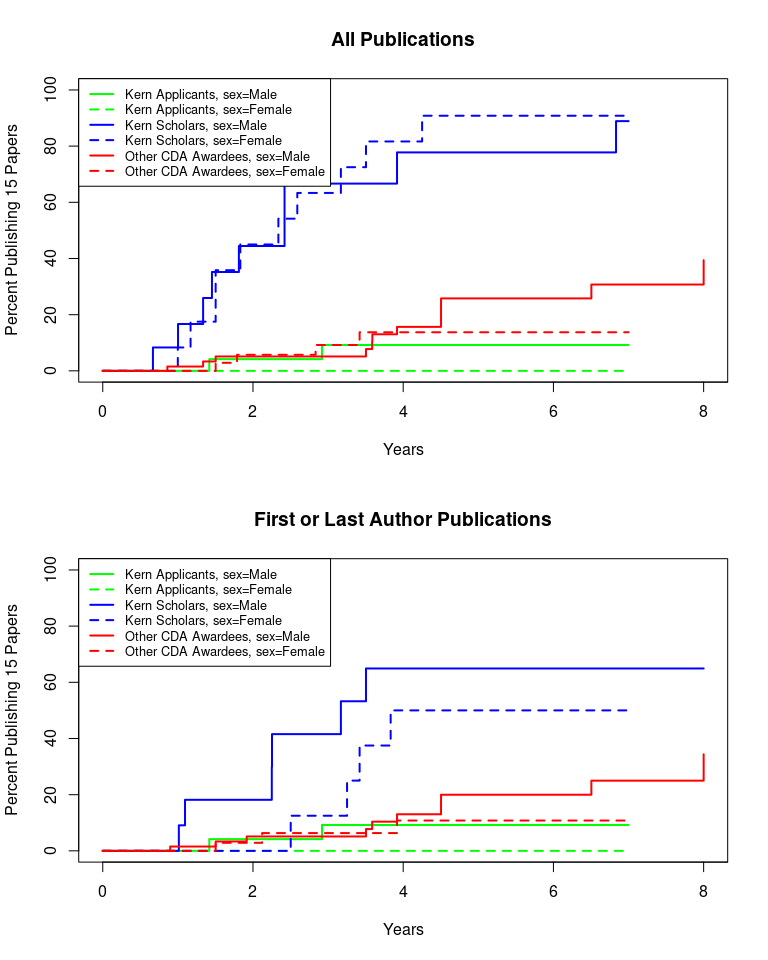


**Figure S2. Time to 15^th^ new publication stratified by race (white vs non-white).**

**Upper panel:** Time to 15^th^ new publication between Kern Scholars and Kern applicants or other CDA awardees.

**Lower panel:** Time to 15^th^ new first-/last-author publication between Kern Scholars and Kern applicants or other CDA awardees.


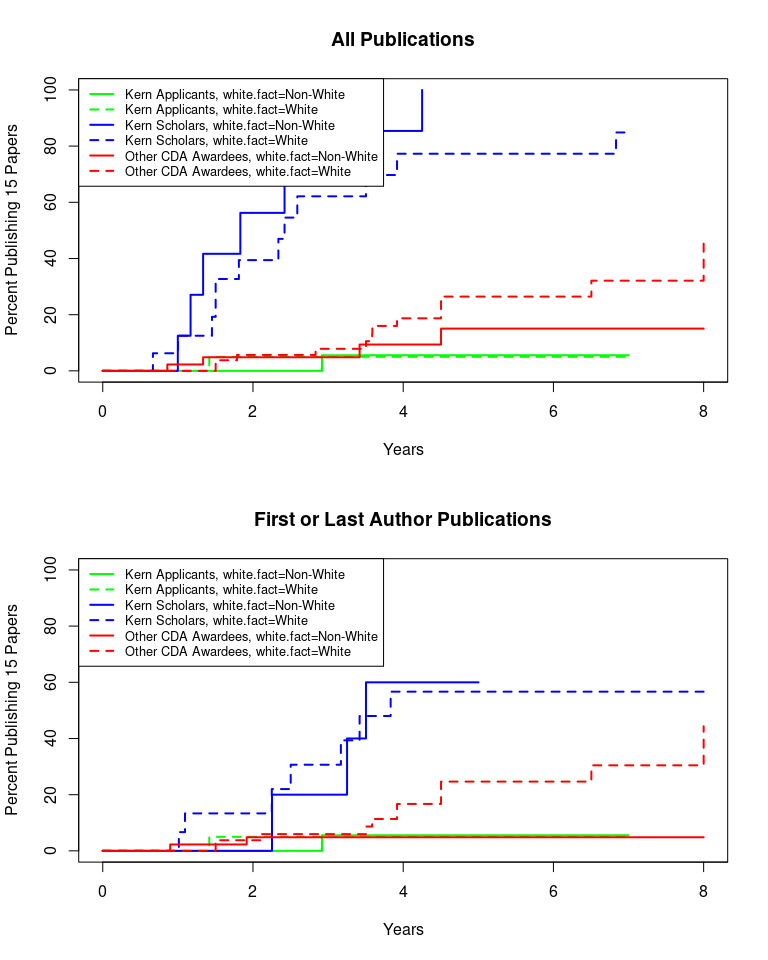


**Figure S3. Time to 15^th^ new publication stratified by specialty (surgical vs non-surgical).**

**Upper panel:** Time to 15^th^ new publication between Kern Scholars and Kern applicants or other CDA awardees.

**Lower panel:** Time to 15^th^ new first-/last-author publication between Kern Scholars and Kern applicants or other CDA awardees.


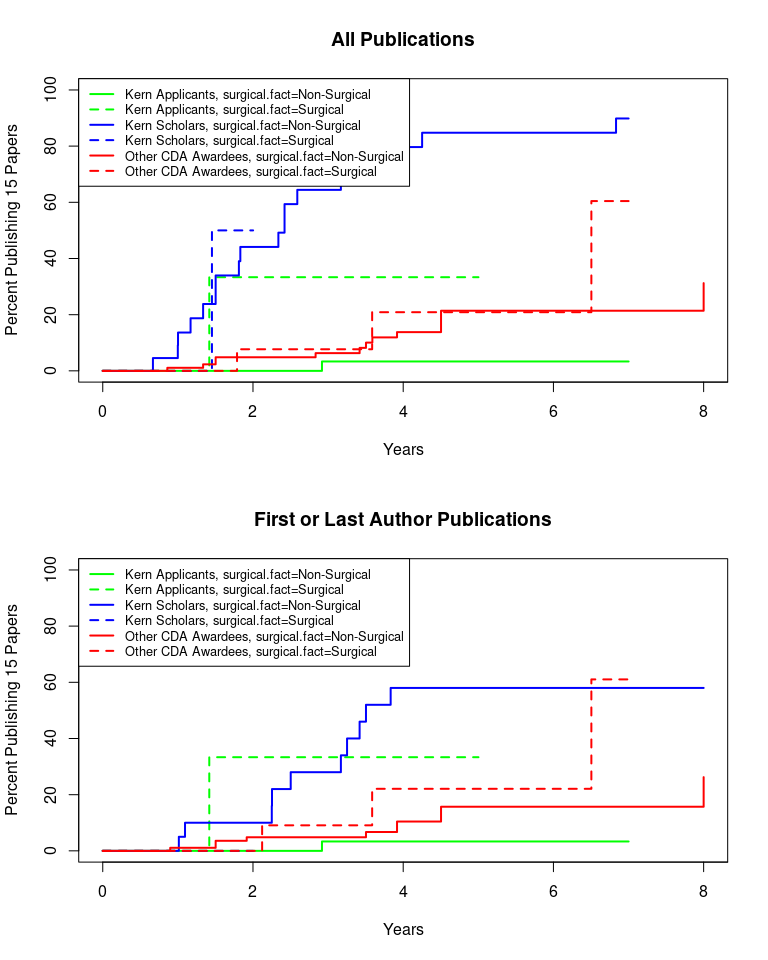

Supplement: Supplementary file 1 [file S2059866121007809sup001.docx]
